# Discovery of Novel GPVI Receptor Antagonists by Structure-Based Repurposing

**DOI:** 10.1371/journal.pone.0101209

**Published:** 2014-06-27

**Authors:** Lewis Taylor, Sridhar R. Vasudevan, Chris I. Jones, Jonathan M. Gibbins, Grant C. Churchill, R. Duncan Campbell, Carmen H. Coxon

**Affiliations:** 1 Department of Physiology, Anatomy and Genetics, University of Oxford, Le Gros Clark Building, South Parks Road, Oxford, United Kingdom; 2 Department of Pharmacology, University of Oxford, Mansfield Road, Oxford, United Kingdom; 3 Institute for Cardiovascular and Metabolic Research, School of Biological Sciences, University of Reading, Hopkins Building, Reading, United Kingdom; University Hospital Medical Centre, Germany

## Abstract

Inappropriate platelet aggregation creates a cardiovascular risk that is largely managed with thienopyridines and aspirin. Although effective, these drugs carry risks of increased bleeding and drug ‘resistance’, underpinning a drive for new antiplatelet agents. To discover such drugs, one strategy is to identify a suitable druggable target and then find small molecules that modulate it. A good and unexploited target is the platelet collagen receptor, GPVI, which promotes thrombus formation. To identify inhibitors of GPVI that are safe and bioavailable, we docked a FDA-approved drug library into the GPVI collagen-binding site *in silico*. We now report that losartan and cinanserin inhibit GPVI-mediated platelet activation in a selective, competitive and dose-dependent manner. This mechanism of action likely underpins the cardioprotective effects of losartan that could not be ascribed to its antihypertensive effects. We have, therefore, identified small molecule inhibitors of GPVI-mediated platelet activation, and also demonstrated the utility of structure-based repurposing.

## Introduction

Cardiovascular disease (CVD) is a leading cause of mortality worldwide accounting for ∼1 in 3 deaths in industrialised societies [1], [2]. A number of antiplatelet therapies are currently approved for the management of pathological thrombosis, including cyclooxygenase (COX) inhibitors (e.g. aspirin), phosphodiesterase (PDE) inhibitors (e.g. cilostazol), irreversible ADP receptor antagonists (e.g. clopidogrel, prasugrel) and fibrinogen receptor blocking antibodies (e.g. abciximab, eptifibatide). Current antiplatelet drugs, by means of their methods of action, have undesirable side effects including haemorrhage (gastrointestinal and cerebral), neutropenia, headache, skin irritation and hypertension. The development of new antiplatelet agents, especially drug-like small molecules, is long overdue [3]–[5].

Drug repurposing is a low-risk, high-gain strategy for drug discovery and development in which drugs used for one indication are repurposed to treat another [6], [7]. This approach reduces the duration, cost and associated risks inherent in traditional drug discovery. Drug repurposing is well and truly in the spotlight and is a major focus for the pharmaceutical industry, the U.S. National Institutes of Health and the UK's Medical Research Council [8], [9]. The application of computational approaches to drug discovery (e.g. virtual screening, *in silico* docking) greatly increases the power of lead identification and the elucidation of novel chemical scaffolds [10]–[12]. Yet despite the power and promise of *in silico* repurposing, such an approach has not so far been reported for antiplatelet agents.

A sensible place to identify new targets for the development of novel therapies is in the physiological processes that underlie the disease. Our most successful antiplatelet agents to date (aspirin and clopidogrel) work by directly inhibiting enzymes and receptors that mediate the secondary phase of platelet activation. However, as previously mentioned, undesirable side effects (inappropriate bleeding) is a major problem with current antiplatelet agents; new targets could yield better drugs with improved efficacy and reduced side effects.

There is evidence to suggest that inhibition of adhesion receptors such as the collagen receptor, GPVI, or the von Willebrand receptor, GPIb-IX-V, may be a viable approach for reducing pathological thrombus formation *in vivo* [*13]*, [*14*]. Platelets adhere to exposed collagen fibres following injury of the vessel wall (physiological response) or rupture of an atherosclerotic plaque (pathological response). Circulating von Willibrand factor (vWF) complexes with exposed collagen fibres and together these proteins act as ligands for the platelet adhesion/activation receptors α2β1, the GPIb-IX-V complex, and glycoprotein (GP) VI [15], [16]. Adhesion via α2β1 and the GPIb-IX-V mediates platelet rolling and tethering, allowing the major platelet collagen receptor, GPVI, to interact with its ligand to mediate platelet activation. Once engaged, GPVI initiates tyrosine kinase-dependent signalling via the associated FcRγ chain, which contains an immunoreceptor tyrosine-based activation motif (ITAM). Ca^2+^ release and subsequent granule secretion leads to the release of secondary mediators of platelet activation, including 5-HT and ADP, that act in an autocrine fashion. Concurrent with these events is the generation of thrombin and fibrinogen, which leads to the formation of a fibrin clot.

There are many mediators of platelet activation that could be targeted for drug development. GPVI activation is an early event in collagen-induced thrombus formation and is receiving increasing attention as a potential target for antiplatelet development. Recent characterisation of GPVI-Fc fusion proteins that block platelet-collagen interactions [17], [18], as well as the development of humanised murine GPVI scFvs [19], have highlighted GPVI as a therapeutic target. GPVI-Fc fusion proteins inhibit collagen responses in animal models, demonstrating a lack of toxicity in human trials, and no bleeding phenotype. The effectiveness of these biologicals in reducing thrombosis is reminiscent of endogenous mechanisms that regulate GPVI-collagen interactions through receptor shedding at high shear [20]. These data strengthen the argument for targeting the collagen-GPVI interaction for therapeutic intervention. However, administration of scFvs, antibodies or fusion proteins relies on intravenous injection and is therefore not a viable approach for self-administration or long-term use outside the clinic, especially for a highly prevalent disease. Development of orally bioavailable small, drug-like, GPVI-specific antagonists could, therefore, be of great therapeutic value [13], [14] as they would provide an alternative option for the management of thrombotic risk. In this study we describe the identification and characterisation of two selective GPVI receptor antagonists *in vitro*.

## Materials and Methods

### Materials

4G10 anti-phosphotyrosine monoclonal antibody was purchased from Millipore (Upstate, U.K.). Losartan, cinanserin and U46619 were purchased from Tocris Bioscience (Bristol, U.K.). All other reagents were purchased from Sigma (Poole, U.K.). The NIH Clinical Collection (1 and 2) was purchased from Evotec (CA, U.S.) Inc.

### *In silico* docking

The crystal structure of human platelet glycoprotein VI (PDB ID 2gi7) [21] was used for *in silico* docking. The receptor was processed by addition of protons and flipping of Gln, His and Asn using MolProbity [22]. The receptor was further processed using the program FRED receptor 2.2.5 [23] and a requirement for H-bonding of Lys 41 with the compounds was set. The docking was performed using Fred 2.2.5 and a database of 727 compounds with conformers pre-generated using Omega 2.2.3 [24]. The molecules were docked and scored with FRED's default consensus scoring and the top 40 molecules were tested for their biological efficacy using a CRP-XL-induced Ca^2+^ release assay.

### Isolation of human platelets

Whole blood was taken from healthy volunteers (following written consent) and collected into 50 ml syringes containing 5 ml 4% sodium citrate in accordance with procedures approved by the Local Research Ethics Committee (Milton Keynes Ref: 07/Q1603/17). Platelet-rich plasma (PRP) was isolated by centrifugation at 200×g for 10 minutes at room temperature. PRP was pooled and 10 μg PGI_2_ was added before centrifugation at 1000×g for 10 minutes at room temperature. Platelets were resuspended in 1ml Tyrodes buffer (134 mM NaCl, 0.34 mM Na_2_HPO_4_, 2.9 mM KCl, 12 mM NaHCO_3_, 20 mM 4-(2-hydroxyethyl)-1-piperazineethanesulfonic acid (HEPES), 5 mM glucose, 1 mM MgCl_2_, pH 7.3) pre-warmed to 30°C and 150 μl ACD. The volume was adjusted to 25 ml with Tyrodes buffer, followed by addition of 3 ml ACD and 1.25 μg PGI_2_. Cells were centrifuged at 1000×g for 10 minutes at room temperature and the resultant cell pellet was resuspended in pre-warmed Tyrodes buffer to a final cell density of 4×10^8^ cells/ml (aggregations) or 2×10^9^ cells/ml (peptide pull downs). Where appropriate, 1 mM ethylene glycol tetraacetic acid (EGTA), 10 μM indomethacin and 2 U/ml apyrase were added to inhibit platelet aggregation (referred to as non-aggregating conditions).

### Light transmission aggregometry

Platelets (450 μl) were stimulated with agonist in a final volume of 500 μl at 37°C with continuous stirring (1200 rpm) in an optical aggregometer. For drug studies, platelets were incubated with losartan or cinanserin for 60 s after which time, agonist was added and aggregations monitored using AGRO/LINK8 software (Chrono-log Corp., Pennsylvania, U.S.A).

### *In vitro* assessment of Ca^2+^ release

Washed human platelets were incubated with 3 μM fura-2 AM for 1 hour at 30°C before being washed once in Tyrodes buffer and resuspended at 4×10^8^ cells/ml. Changes in fluorescence were measured in a BMG Fluostar Optima plate reader using excitation wavelengths of 340 nm and 380 nm. [Ca^2+^]i was calculated using the following formula: [Ca^2+^]i =  Kd × (Rmin)/(Rmax –R) × Sfb, where K_d_ (for Ca^2+^ binding to fura-2 at 37°C)  = 225 nM, R = 340/380 ratio, Rmax  = 340/380 ratio under Ca^2+^-saturating conditions, Rmin  = 340/380 ratio under Ca2+-free conditions, and Sfb  =  ratio of baseline fluorescence (380 nm) under Ca^2+^-free and -bound conditions [25]. For drug studies, compounds were pre-incubated with the platelets for 2 minutes at 37°C with orbital shaking before the addition of agonist.

### Western blotting

Samples were boiled in Laemmli buffer (working concentration 50 mM Tris-HCl, pH 6.8, 100 mM dithiothreitol (DTT), 2% sodium dodecyl sulfate (SDS), 0.01% bromophenol blue, 10% glycerol). Proteins were resolved on NuPAGE pre-cast Tris-Glycine gels (Invitrogen, U.K.) prior to transfer onto nitrocellulose at 70 mA per gel for 40–60 min. Membranes were blocked in 5% non-fat milk powder/Tris-buffered saline/0.1% Tween-20 (TBST; 50 mM Tris, 150 mM NaCl, 0.1% Tween-20) with gentle agitation for one hour at room temperature (RT) or overnight at 4°C. Primary antibody was added to the membranes at the appropriate dilution in 5% Marvel/TBST with gentle agitation for 1.5 h at RT or overnight at 4°C. Membranes were washed 3 times in 1× TBST for 10 minutes. The appropriate HRP-conjugated secondary antibody (DAKO) was added to the membranes in 5% non-fat milk powder/TBST and incubated at room temperature for 45 min with gentle agitation. Membranes were washed 3 times in 1× TBST for 10 min. Bands were then visualised with ECL Plus detection reagent (GE Lifesciences, Little Chalfont, U.K.) using an AGFA Curix developer.

### Flow cytometry

#### P-selectin and FITC-fibrinogen binding

Whole blood (5 µl) was incubated with 2 µl anti-CD62P-phycoerythrocynin (PE) conjugates antibody (BD Bioscience, UK) and 2 µl fluorescein isothiocyanate (FITC)-fibrinogen (DAKO, Ely, U.K.), drugs and agonist, in a final volume of 50 µl Tyrodes-HEPES buffer. To this, a CRP-XL was added to a final concentration of 1 µg/ml and incubated at room temperature for 10 minutes. Samples were then fixed in 2 ml 0.2% formyl saline (sterile filtered). Exposure of P-selectin (CD62P) and FITC-fibrinogen binding was measure using a BD Accuri C6 flow cytometer. *GPVI levels*: Whole blood (5 µl) was incubated with drug for 10 minutes at room temperature. To this, 2 µl anti-GPVI antibody (GPVI (HY101, M.L. Kahn, University of Pennsylvannia, 1 mg/ml) was added and incubated for 10 mins at room temperature. Donkey anti-mouse antibody conjugated to Alexa Fluor 647 was added to the samples and incubated at room temperature for a further 10 minutes. Samples were then fixed in 2 ml 0.2% formyl saline (sterile filtered) and read on a BD Accuri C6 flow cytometer.

## Results

### *In silico* docking yields high hit rate and streamlines screening

**Figure 1:**
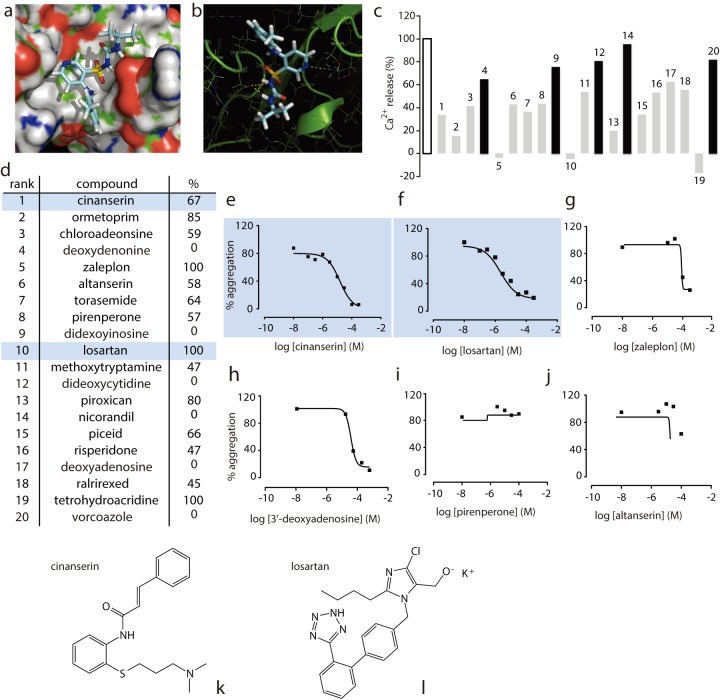
*In silico* identifies GPVI antagonists. Representative image capture of *in silico* docking into GPVI using Glide, with space filling model is shown in a, and H-bonding to relevant side chains is detailed in b. The 20 highest ranking compounds were screened for effects on Ca^2+^ release by the GPVI-specific agonist CRP-XL (10 µg/ml) (c and d, % refers to percent inhibition of Ca^2+^ release). Maximum Ca^2+^ release is shown in white, compounds that inhibited Ca^2+^ release by ∼50% or more are in grey, and the remainder in black. Commercially available compounds that inhibited CRP-XL-induced Ca^2+^ release >50% were further screened by light transmission aggregometry to identify compounds displaying dose-dependent inhibition (e-j). Examples are shown of weak antagonism (g and h) and false positives (i and j). Cinanserin (l) and losartan (k) were taken on for further study.

A library of 727 FDA-approved drugs and their diastereomers were docked into the GPVI ligand binding site, with an emphasis on the electrostatic environment at the Lys41 position (Figure 1a and 1b). Lys41 lies within the floor of the groove that forms the collagen binding site of GPVI [21], [26], [27] and compounds were ranked on their ability to bind in this region. Using a single point screen (Ca^2+^ release) with a high dose of drug (100 μM) and a high dose of cross-linked collagen related peptide (CRP-XL, 10 μg/ml), we found that docking yielded a high success rate; of the top 20 compounds, 14 inhibited CRP-XL-induced Ca^2+^ release from fura2-AM-loaded washed human platelets by ≥50% (Figure 1c and 1d). Then, using light transmission aggregometry over a limited (5–6 point) 3-fold dilution series from 100 µM to 300 nM, we re-screened those drugs that had demonstrated inhibition of Ca^2+^ release and were commercially available, to identify those which gave concentration-dependent inhibition of GPVI-mediated aggregation (Figure 1e-k). Of these drugs, some were false positives (e.g. altanserin and pirenpirone – Figure 1i and j) while others showed only weak antagonism (e.g. zaleplon and deoxyadenosine – Figure 1g and h). Cinanserin and losartan both demonstrated concentration-dependent inhibition with suitable IC_50_ values (Figure 1e and f) and were therefore selected for further investigation.

Cinanserin (Figure 1l) was the highest ranking hit (#1) from the docking and preliminary experiments indicated that it had an IC_50_ in the micromolar range. The angiotensin II type I receptor antagonist losartan (Figure 1m) also ranked highly as a potential antagonist (#10). Retrospective literature searches revealed that this compound had previously been reported to interact with the collagen binding pocket of GPVI [28] and inhibit platelet aggregation both *in vitro* [29] and *in vivo* [30]. The fact that losartan has been shown to interact with GPVI at the collagen binding site in an independent study validates our methodology and provides confidence that our docking parameters are appropriate. Losartan was included in subsequent experiments as it serves not only as a positive control, but also as a potential drug for repurposing if found to be selective and efficacious; losartan is a well-tolerated drug. Cinanserin was also included for further characterisation as it was ranked with the greatest probability of interacting with GPVI, and exhibited robust inhibition of Ca^2+^ release (Fig. 1c and 1e). The high success rate of our computational screen (14 out of 20 compounds inhibited CRP-XL-induced Ca^2+^ release *in vitro*) validates the use of an *in silico* docking strategy for identifying potential receptor antagonists.

### Losartan and cinanserin inhibit Ca^2+^ release and aggregation in washed human platelets

**Figure 2:**
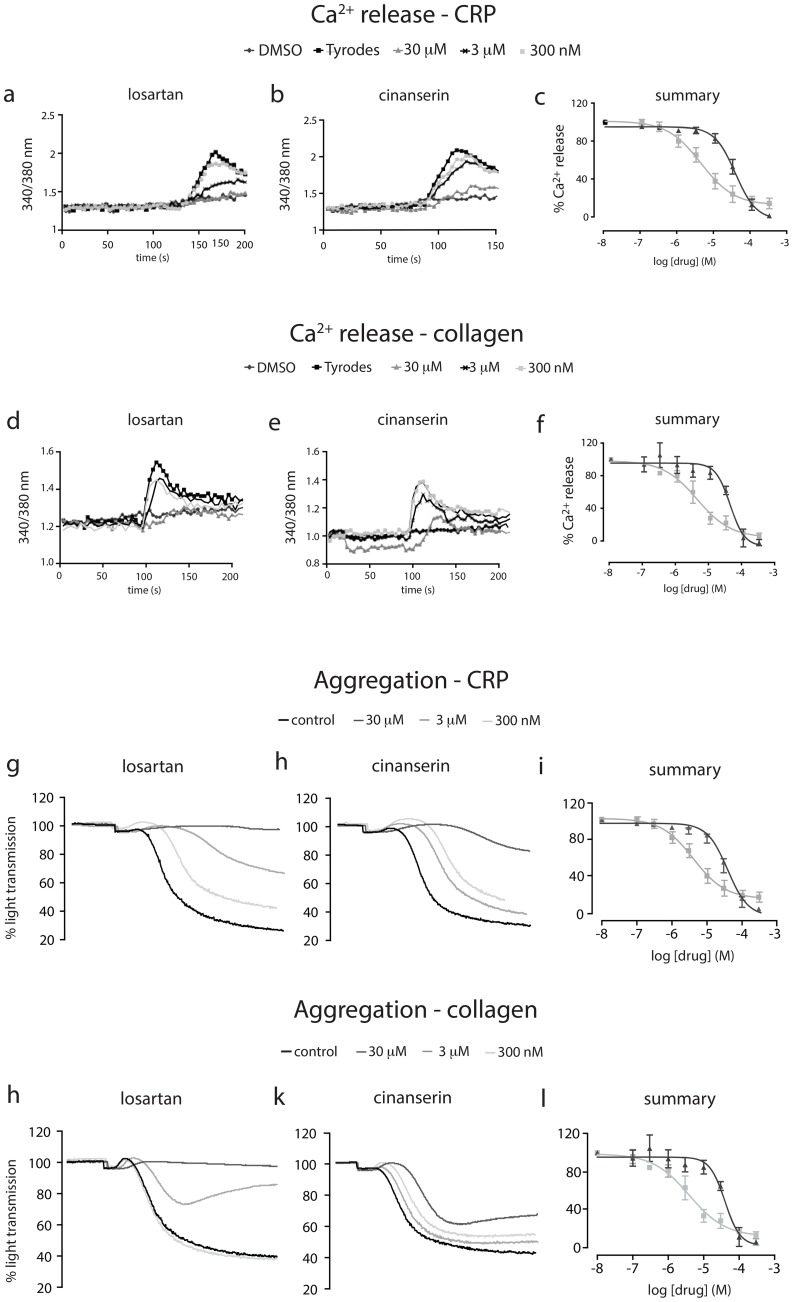
Losartan and cinanserin inhibit GPVI-mediated cell activation. Washed human platelets were loaded with fura2-AM and screened for drug-mediated inhibition of Ca^2+^ release by 1 µg/ml CRP-XL (n = 3, ± SEM, representative traces and summary, a–c) and 1 µg/ml collagen (n = 3, SEM, representative traces and summary, d–f); losartan (▪) and cinanserin (▴). To measure aggregation, washed human platelets were incubated with drug for one minute prior to the addition of 1 μg/ml CRP-XL (representative traces and summary shown in g–i) or 1 µg/ml collagen (representative traces and summary shown in j–l).

We next conducted more detailed studies to determine precise IC_50_ values for losartan and cinanserin using platelet functional assays. Ca^2+^ release induced by the GPVI-specific agonist CRP-XL, and the endogenous ligand collagen, was measured and quantified. Both drugs inhibited Ca^2+^ release in a concentration-dependent manner in response to both CRP-XL (Figure 2a–c) and collagen (Figure 2d–f). Cinanserin and losartan had IC_50_ values in the micromolar range, with losartan having an IC_50_ value of 4 µM, 10-fold greater than that of cinanserin (40 µM). To determine whether the inhibitory effect of losartan and cinanserin on Ca^2+^ release could be translated into effects on gross platelet function, we carried out light transmission aggregometry using washed human platelets. Both drugs exhibit dose-dependent inhibition of platelet aggregation in response to both CRP-XL (1 µg/ml, Figure 2g–i) and collagen (1 µg/ml, Figure 2j–l). IC_50_ values were comparable to those for Ca^2+^ release and are summarised in Table 1.

**Table 1:** IC_50_ values for losartan and cinanserin on both CRP-XL- and collagen-induced Ca^2+^ release and aggregation.

	Ca^2+^ release		Aggregation	
	CRP	Collagen	CRP	Collagen
**losartan**	4	2	4	4
**cinanserin**	40	40	35	40

Washed human platelets were assessed for effects on Ca^2+^ release and aggregation (n = 3–5). Values are in μM.

### Losartan and cinanserin are selective inhibitors

**Figure 3:**
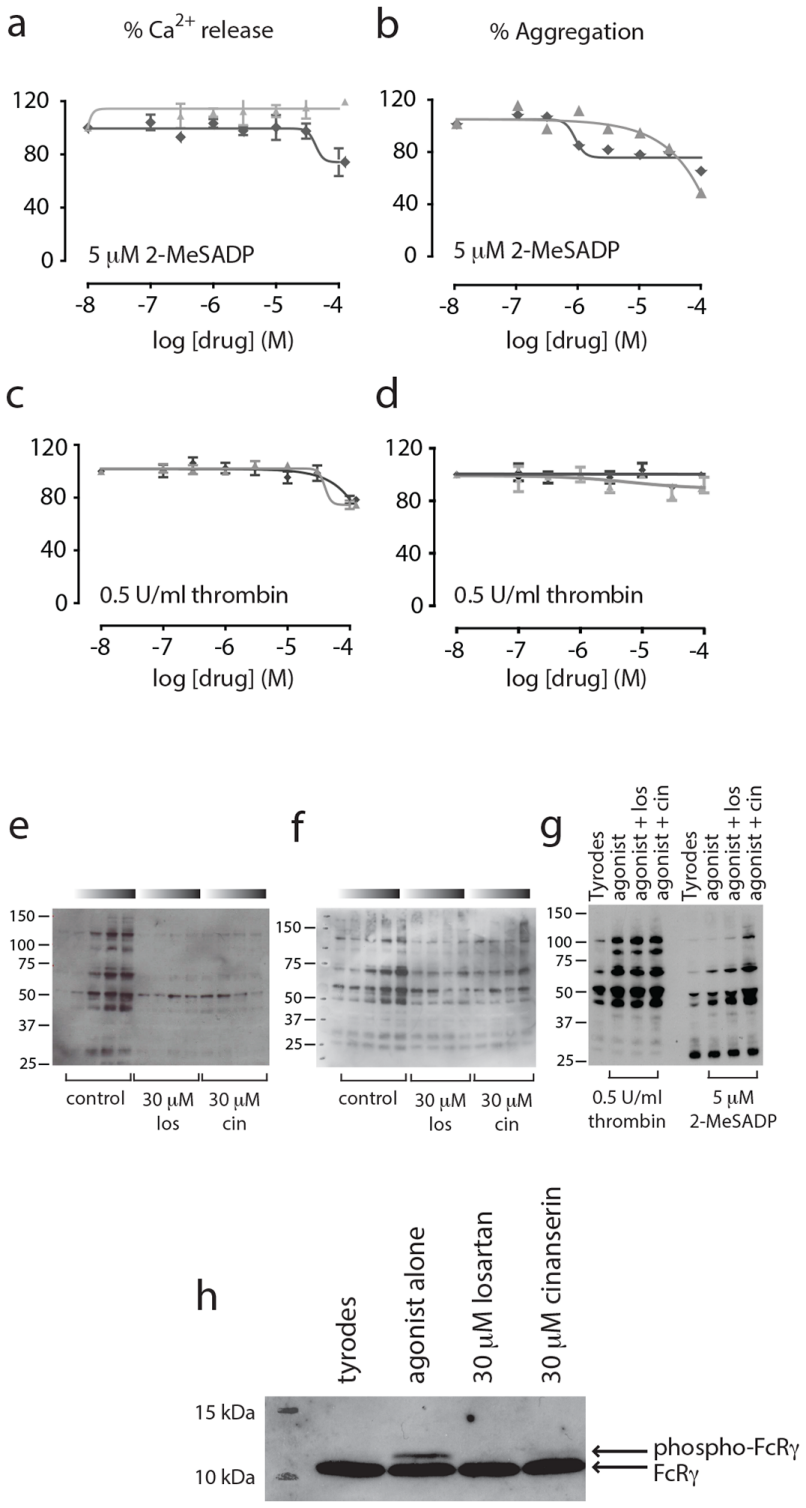
Losartan and cinanserin demonstrate selectivity for GPVI. Ca^2+^ release and aggregations were carried out with 5 μM of the P_2_Y_12_ receptor agonist 2-MeSADP (a, Ca^2+^ release and b, aggregation), or 0.5 U/ml of the PAR1 and PAR4 receptor agonist thrombin (c, Ca^2+^ release and d, aggregation). Losartan (⧫); cinanserin (▴), n = 3, ± SEM. For global tyrosine phosphorylation, washed human platelets were incubated with drug or vehicle alone before addition of 1 µg/ml CRP-XL or collagen. Samples were collected at 10, 30, 60 or 90 seconds (as indicated by the graduated bars with time increasing to the right) in ice cold 2× lysis buffer and separated on 4–12% NuPage gels under reducing conditions. Tyrosine phosphorylation was visualized with 4G10 anti-phosphotyrosine antibody. Losartan and cinanserin reduce CRP-XL- (e) and collagen-(f) induced global tyrosine phosphorylation, but have no effect on thrombin or 2-MeSADP induced global tyrosine phosphorylation (g). Both drugs reduce FcRγ phosphorylation (h), (unphosphorylated, lower band; phosphorylated, upper band).

Losartan and cinanserin inhibit CRP-XL- and collagen-induced Ca^2+^ release and aggregation. To experimentally determine the selectivity of these compounds we tested their ability to antagonise other platelet agonists. Ca^2+^ release and aggregation responses to the P2Y_12_ receptor agonist 2-MeSADP (5 μM), and the PAR1/4 agonist thrombin (0.5 U/ml), in the presence and absence of drug, were measured. Losartan and cinanserin had minimal effects on P2Y_12_–mediated platelet activation, although some high concentration effects (at 100 µM) were observed for MeSADP-induced Ca^2+^ release (Figure 3a) and aggregation (Figure 3b). With regards to thrombin, little or no effect is seen for both Ca^2+^ release (Figure 3c) or aggregation (Figure 3d).

Engagement of GPVI with its ligand induces tyrosine phosphorylation of the associated FcRγ chain on conserved tyrosine residues within its ITAMs [31] by Src family kinases that are non-covalently associated with the receptor. This phosphorylation leads to recruitment and activation of the tyrosine kinase Syk, which mediates phosphorylation of a number of downstream targets leading to an increase in global tyrosine phosphorylation [32]. Both losartan and cinanserin reduced global tyrosine phosphorylation induced by the GPVI-specific agonist CRP-XL (Figure 3e), and the endogenous ligand collagen (3f). No effect on either thrombin- or 2-MeSADP-induced global tyrosine phosphorylation (90 s) was observed (Figure 3g). In addition, incubation of drug (30 µM) prior to the addition of CRP-XL reduced phosphorylation of the FcRγ chain (Figure 3h), indicating that losartan and cinanserin prevent phosphorylation of the ITAM and initiation of phosphotyrosine signalling following ligand engagement.

**Figure 4:**
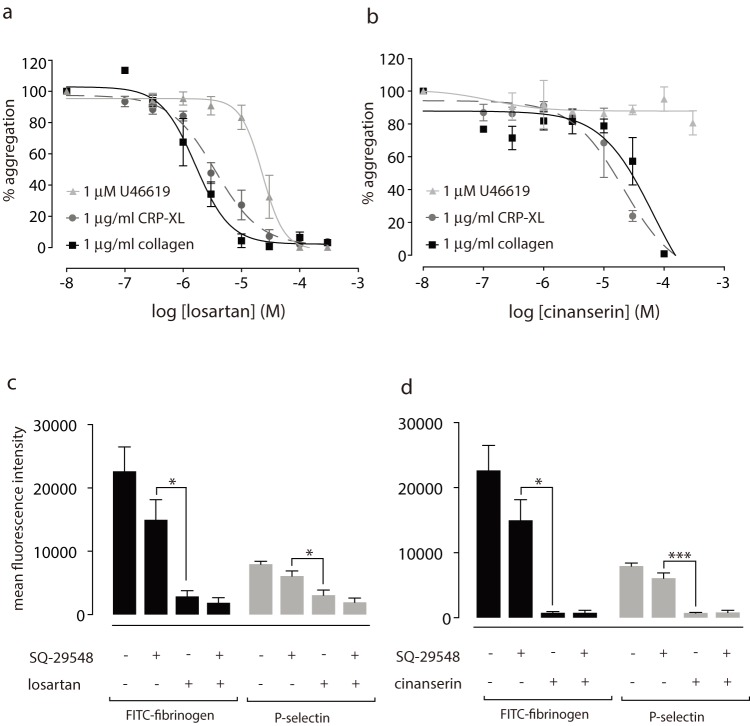
Losartan is selective for GPVI over TPR. To assess effects on TPR signalling, platelets were activated with 1 µM U46619 and aggregations followed for 5 minutes in the presence of or absence of drug (n = 3, ± SEM). Collagen (solid black line) and CRP-XL (dashed dark grey line) were both used at 1 µg/ml. Losartan (a) has an IC_50_ of ∼20 µM for U46619-induced aggregation (solid grey line) while cinanserin (b) has no effect on TPR signaling (solid grey bar). Exposure of P-selectin (CD62P) and FITC-fibrinogen binding was measured by flow cytometry. Losartan (100 µM, c) and cinanserin (100 µM, d) both reduce FITC-fibrinogen binding (black bars) and P-selectin exposure (grey bars) compared to SQ-29548 alone. Statistical analysis was conducted by one-way ANOVA with Sidak's multiple comparisons correction. ns P>0.05, * P<0.05, *** P<0.001, n = 3, ± SEM.

Recent studies pertaining to the antiplatelet effects of losartan have focused mainly on its inhibition of thromboxane A_2_ receptor (TPR) signaling [33], [34], rather than collagen. When platelet rich plasma is isolated from mice that have been injected with losartan (10 mg/kg) for 5 days, aggregation responses to the TPR agonist, U46619, are inhibited, and these inhibitory effects extend to thrombus formation *in vivo* [30] (FeCl_3_-induced injury model). Yet our studies indicate that losartan is, thus far, relatively selective for GPVI. To address this discrepancy, we examined the effects of losartan and cinanserin on U46619 (1 µM)-induced platelet aggregation in washed human platelets. Losartan (Figure 4a) has an IC_50_ of ∼20 µM for U46619-induced platelet aggregation, compared to ∼2–4 µM for collagen (1 µg/ml) and CRP-XL (1 µg/ml) (Table 1). These results indicate that while losartan inhibits some component of TP receptor signalling, its effects on the GPVI receptor are more potent. Cinanserin had no effect on U46619-mediated aggregation (Figure 4b).

To determine the relative contribution of TPR signalling to the inhibitory effects of losartan, we measured P-selectin (CD62P) exposure and FITC-fibrinogen binding in whole human blood in the presence or absence of the TPR-specific antagonist, SQ-29548 (50 nM). Cinanserin was included as a control as this drug has no effect on U46619-induced platelet activation (Figure 4b). Losartan significantly reduced FITC-fibrinogen binding, and P-selectin exposure, beyond that of SQ-29548 alone (Figure 4c). Adding both compounds together results in a level of inhibition similar to that of losartan alone. Cinanserin also significantly reduced FITC-fibrinogen binding and P-selectin exposure when compared to SQ-29548 alone (Figure 4d). This data shows that the inhibitory effects of losartan on GPVI-mediated platelet activation are not due solely to effects on TPR. There is an additional inhibitory effect on CRP-XL-induced platelet activation above and beyond its effects on TPR that similar to that of cinanserin.

### GPVI antagonists compete for ligand binding

**Figure 5:**
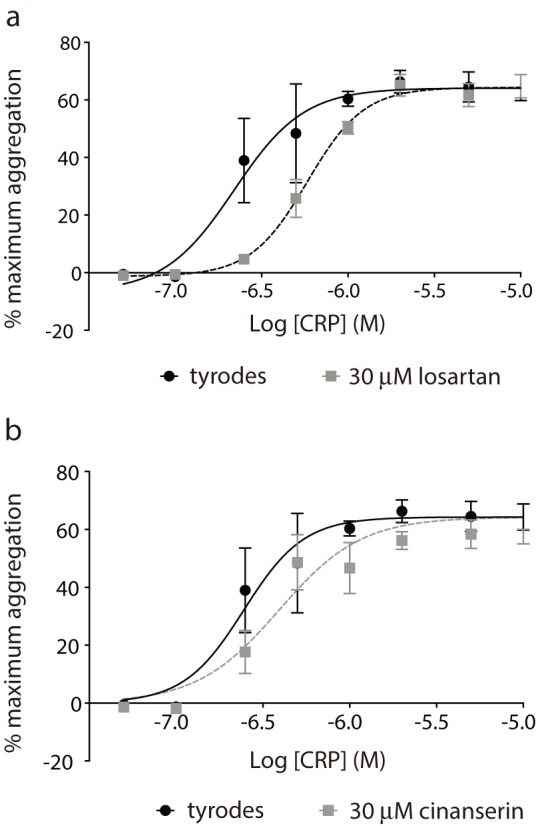
Losartan displays characteristics of competitive antagonists. Platelet aggregation responses to the GPVI-specific agonist CRP-XL were determined to a range of concentrations in the presence or absence of 30 µM drug. Losartan (a) significantly reduced the EC_50_ of CRP (F_(1,58)_ = 15.79, p = <0.001). Cinanserin (b) also reduced the EC_50_ of CRP (F_(1,60)_ = 4.07, p = 0.048).

From the data presented above, losartan and cinanserin demonstrate selectivity for inhibition of GPVI-mediated platelet activation. For a small molecule pharmacological agent, a competitive mechanism of action is preferable [35], so we set out to determine whether our antagonists act in a competitive manner. Platelet responses to CRP-XL were measured over a range of concentrations in the presence or absence of 30 µM drug (Figure 5). Losartan caused a rightward shift of the curve compared to vehicle alone, and increased the EC_50_ value for CRP-XL 3-fold (n = 4, p = 0.0008). Minima and maxima values are equal to that of the control (Figure 5a), indicative of competitive binding. Cinanserin also caused a rightward shift of the curve and increased the EC_50_ of CRP-XL by 2-fold (n = 4, p = 0.0487) suggesting it also acts in a competitive manner (Figure 5b). We next measured levels of GPVI at the cell surface by flow cytometry to experimentally verify that that the inhibitory effects of losartan and cinanserin were not due to drug-mediated decreases in receptor levels; neither drug affected GPVI levels (n = 3).

### GPVI antagonism by losartan is unique amongst the sartan family

**Figure 6:**
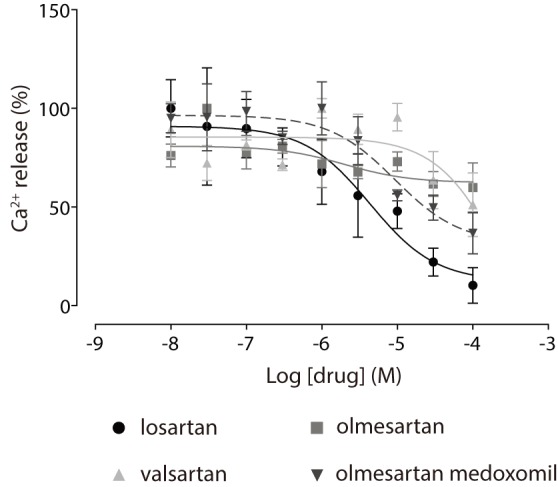
GPVI antagonism is unique to losartan amongst the sartan drug class. Inhibition of collagen-induced Ca^2+^ release (10 µg/ml) the sartans from fura2-AM-loaded washed human platelets was measured and quantified (n = 3, ± SEM).

Losartan is one of a class of angiotensin receptor blockers (ARBs) that also includes valsartan and olmesartan, amongst others. Valsartan does not have the protective effects on cardiovascular outcomes reported for losartan *in vivo* [*36*], suggesting that the inhibitory effects of losartan on GPVI may be unique amongst this drug class, and there is structural evidence to support this [28]. Three sartans were compared with losartan for effects on GPVI-mediated Ca^2+^ release to determine whether GPVI antagonism is shared amongst this drug class. Losartan was the most potent and efficacious inhibitor of collagen-induced Ca^2+^ release (10 µg/ml collagen, n = 3, Figure 6), with some activity observed for the pro-drug olmesartan medoxomil.

## Discussion

The current therapeutic strategy for managing the occurrence of arterial thrombosis generally relies on dual antiplatelet therapy (thienopyridines and aspirin). This approach is cost-effective and reduces thrombotic risk, but comes at a price, with a considerable number of patients experiencing bleeding complications. Genetic polymorphisms can also reduce the effectiveness of both aspirin [37]–[39] and the thienopyridines [40] resulting in drug ‘resistance’ in ∼20–30% of patients [41].

GPVI is one of a number of potential targets that have been proposed for the development of new antiplatelet agents [13]. Soluble GPVI-Fc fragments (Revacept) can reduce platelet adhesion and recruitment at sites of vascular injury while having only moderate effects on tail bleeding times in mice [42]. In human phase I clinical trials, Revacept had no adverse effects on health or bleeding time, with platelets showing reduced responses to collagen, but not to ADP or thrombin, *ex vivo* (similar to our observations for losartan and cinanserin *in vitro*). Although Revacept may protect against pathological thrombosis, administration requires recurrent intravenous injection and patients cannot self-administer at home. In addition, continued administration over prolonged periods may lead to the generation of antibodies by the host immune system that neutralise the fusion protein. The provision of orally available small molecule drugs that inhibit GPVI interactions with exposed collagens may provide a new clinical route for the long-term management of recurrent arterial thrombosis.

To find such drugs, we adopted a drug repurposing strategy to identify new GPVI receptor antagonists from a library of FDA-approved drugs. Using this unbiased approach, we identified compounds that demonstrated GPVI antagonism and selectivity with IC_50_ values in the micromolar range. Losartan is a generic FDA-approved compound for the treatment of hypertention, while cinanserin is a 5-HT receptor antagonist which, although not used in clinical practice for decades, has recently attracted attention as an inhibitor of SARS coronoavirus replication [43], [44]. Neither of these compounds are associated with bleeding complications, but, retrospectively, both had been reported to have effects on platelet function. In addition, losartan had been shown to interact with GPVI at the collagen binding site at/around Lys41, by NMR [28]. Due to previous observations that losartan can affect TPR signaling [30], we looked for effects on TPR-mediated platelet activation by the agonist U46619. Losartan inhibited TPR signaling with an IC_50_ value ∼10-fold higher than for GPVI. In addition, losartan (and cinanserin) reduce FITC-fibrinogen binding and P-selectin exposure in response to CRP-XL when TPR is inhibited by SQ-29548. This inhibition is much greater than that of SQ-29548 alone, demonstrating that both losartan and cinanserin have effects on CRP-XL-induced platelet aggregation not attributable to TPR. It is, therefore, reasonable to say that based on our studies losartan is not *specific* for GPVI, but displays selectivity for this receptor in washed human platelets and whole blood experiments. Most drugs lack total specificity and hit more than one target, and in this case, targeting both the GPVI and the TP receptor may actually prove beneficial in terms of antiplatelet activity. Indeed, the TP receptor antagonist terutroban showed promise as an antiplatelet in phase II clinical trials, but was found to offer no advantage over aspirin [5], [45]–[47] with regards to bleeding risk [46]. Cinanserin appears to be more selective for GPVI than losartan, but its IC_50_ was high (∼40 µM) and it is unlikely to reach therapeutic levels in the blood. It may serve as a starting point for future drug design but in terms of drug repurposing it s not a viable candidate.

Losartan may hold more promise in terms of immediate clinical application as an antiplatelet agent. This well-tolerated antihypertensive undergoes conversion (∼14%) to a metabolite (EXP3174) that is 10–40-fold more potent than the parent drug. Both compounds act at the ATII type I receptor to antagonise ATII-mediated effects *in vivo*. A recent study demonstrated that mice administered daily injections of losartan for 5 days exhibit reduced platelet aggregation in response to U46619 *ex vivo*, implicating losartan as a TP receptor antagonist. Mice also demonstrate reduced thrombus formation *in vivo* (pathological), but not tail bleeding times (physiological). It is interesting to note that these *in vivo* outcomes are similar to that of Revacept (a soluble GPVI-Fc fragment), which also reduces thrombus formation *in vivo* while having minimal effects on physiological bleeding responses. However, the authors of this study did not examine CRP-XL- or collagen-induced signaling. The antagonistic effects on GPVI signalling of losartan appears to be unique amongst the sartan drug class – some activity is seen for the pro-drug olmesartan medoxomil, but rapid conversion to its active form (olmesartan), which does not antagonise GPVI-mediated Ca^2+^ release, is unlikely to exert GPVI antagonism *in vivo*. It is likely that the anti-platelet effects of losartan can be attributed to inhibition of both GPVI and TP signalling. Data from the Losartan Intervention For Endpoint reduction (LIFE) and follow-up studies linked losartan with a reduction in cardiovascular mortality (stroke and myocardial infarction) [48]–[50], and improved clinical outcomes. The authors of the 2002 LIFE study state ‘losartan seems to have benefits beyond blood pressure reduction’ [48], and it is tempting to speculate that this could be due to its antiplatelet effects. A similar study of valsartan (VALUE [36]) did not show any effect on cardiovascular outcome, and this may be due to the fact that valsartan does not have the antiplatelet properties and GPVI antagonism that losartan does. With regards to whether losartan is likely to reach plasma concentrations high enough to elicit antiplatelet effects *in vivo*, adults given 50 mg losartan daily for 7 days have Cmax values of the parent drug, and its metabolite, of 224 ng/ml and 212 ng/ml, respectively, equating to a plasma concentration of around 500 nM. The IC_50_ of losartan for CRP or collagen induced platelet aggregation or Ca^2+^ release is around 2–4 μM; a concentration of 500 nM losartan gives around 10–15% inhibition of platelet responses to GPVI agonists in our hands. Losartan can be given at doses of up to 150 mg per day and it is quite possible that, certainly at higher doses, plasma levels will reach concentrations high enough to reduce platelet responses to collagen.

Losartan is already known to convey a 25% relative risk reduction for stroke when compared to the non-ARB antihypertensive agent, atenolol. Aspirin, although the gold standard for antiplatelet therapy is, actually, associated with a slight *increase* in the risk of haemorrhagic stroke. Losartan is, therefore, more efficacious than aspirin for the prevention of stroke, and not reported to have any bleeding effects associated with its clinical use. It is also a generic drug and therefore cost effective. Further to this, losartan, and other ARBs and ACE inhibitors, have been shown to improve outcomes for Alzheimer's Disease patient by improving cerebral blood flow and reducing inflammatory responses [51]. It appears that losartan could offer a broad range of health benefits in an increasingly aging population.

The work described herein supports our hypothesis that perturbation of GPVI-collagen interactions by small molecules can inhibit platelet aggregation, lending considerable weight to the argument for their development as antiplatelet drugs. In summary, we report the identification of two GPVI-selective antagonists that may serve as a basis for future drug design (cinanserin) or repurposing (losartan). Further work will be required to determine the efficacy of losartan as a *bone fide* GPVI inhibitor *in vivo*, but our study sets the scene for a new wave of antiplatelet agents that target GPVI, and a offers a new strategy for the management of cardiovascular risk.

## References

[1] BHF (2009–2010) BHF coronary heart disease statistics. British Heart Foundation Health Promotion Research Group.

[2] Roger VL, Go AS, Lloyd-Jones DM, Benjamin EJ, Berry JD, et al (2012) Heart disease and stroke statistics –2012 update: a report from the American Heart Association. Circulation 125: e2–e220. 22179539 10.1161/CIR.0b013e31823ac046 PMC4440543

[3] Yeung J, Holinstat M (2012) Newer agents in antiplatelet therapy: a review. J Blood Med 3: 33–42. 22792011 10.2147/JBM.S25421 PMC3393068

[4] Michelson AD (2010) Antiplatelet therapies for the treatment of cardiovascular disease. Nat Rev Drug Discov 9: 154–169. 20118963 10.1038/nrd2957

[5] Franchini M, Mannucci PM (2009) New antiplatelet agents: why they are needed. European journal of internal medicine 20: 733–738. 19892299 10.1016/j.ejim.2009.09.005

[6] Ashburn TT, Thor KB (2004) Drug repositioning: identifying and developing new uses for existing drugs. Nat Rev Drug Discov 3: 673–683. 15286734 10.1038/nrd1468

[7] Mizushima T (2011) Drug discovery and development focusing on existing medicines: drug re-profiling strategy. J Biochem 149: 499–505. 21436140 10.1093/jb/mvr032

[8] NIH (2012) Rescuing and Repurposing Drugs. National Institutes of Health.

[9] Huang R, Southall N, Wang Y, Yasgar A, Shinn P, et al (2011) The NCGC pharmaceutical collection: a comprehensive resource of clinically approved drugs enabling repurposing and chemical genomics. Sci Transl Med 3: 80ps16. 10.1126/scitranslmed.3001862 PMC3098042 21525397

[10] Ballester PJ, Mangold M, Howard NI, Robinson RL, Abell C, et al (2012) Hierarchical virtual screening for the discovery of new molecular scaffolds in antibacterial hit identification. J R Soc Interface. 10.1098/rsif.2012.0569 PMC3481598 22933186

[11] Katritch V, Jaakola VP, Lane JR, Lin J, Ijzerman AP, et al (2010) Structure-based discovery of novel chemotypes for adenosine A(2A) receptor antagonists. J Med Chem 53: 1799–1809. 20095623 10.1021/jm901647p PMC2826142

[12] Shoichet BK (2004) Virtual screening of chemical libraries. Nature 432: 862–865. 15602552 10.1038/nature03197 PMC1360234

[13] Stoll G, Kleinschnitz C, Nieswandt B (2008) Molecular mechanisms of thrombus formation in ischemic stroke: novel insights and targets for treatment. Blood 112: 3555–3562. 18676880 10.1182/blood-2008-04-144758

[14] Kleinschnitz C, Pozgajova M, Pham M, Bendszus M, Nieswandt B, et al (2007) Targeting platelets in acute experimental stroke: impact of glycoprotein Ib, VI, and IIb/IIIa blockade on infarct size, functional outcome, and intracranial bleeding. Circulation 115: 2323–2330. 17438148 10.1161/CIRCULATIONAHA.107.691279

[15] Gibbins JM (2004) Platelet adhesion signalling and the regulation of thrombus formation. J Cell Sci 117: 3415–3425. 15252124 10.1242/jcs.01325

[16] Pugh N, Simpson AM, Smethurst PA, de Groot PG, Raynal N, et al (2010) Synergism between platelet collagen receptors defined using receptor-specific collagen-mimetic peptide substrata in flowing blood. Blood 115: 5069–5079. 20351310 10.1182/blood-2010-01-260778 PMC2890152

[17] Ungerer M, Rosport K, Bultmann A, Piechatzek R, Uhland K, et al (2011) Novel antiplatelet drug revacept (Dimeric Glycoprotein VI-Fc) specifically and efficiently inhibited collagen-induced platelet aggregation without affecting general hemostasis in humans. Circulation 123: 1891–1899. 21502572 10.1161/CIRCULATIONAHA.110.980623

[18] Goebel S, Li Z, Vogelmann J, Holthoff HP, Degen H, et al (2013) The GPVI-Fc fusion protein Revacept improves cerebral infarct volume and functional outcome in stroke. PLoS One 8: e66960. 23935828 10.1371/journal.pone.0066960 PMC3720811

[19] Muzard J, Bouabdelli M, Zahid M, Ollivier V, Lacapere JJ, et al (2009) Design and humanization of a murine scFv that blocks human platelet glycoprotein VI in vitro. Febs J 276: 4207–4222. 19558491 10.1111/j.1742-4658.2009.07129.x

[20] Al-Tamimi M, Tan CW, Qiao J, Pennings GJ, Javadzadegan A, et al (2012) Pathologic shear triggers shedding of vascular receptors: a novel mechanism for down-regulation of platelet glycoprotein VI in stenosed coronary vessels. Blood 119: 4311–4320. 22431567 10.1182/blood-2011-10-386607

[21] Horii K, Kahn ML, Herr AB (2006) Structural basis for platelet collagen responses by the immune-type receptor glycoprotein VI. Blood 108: 936–942. 16861347 10.1182/blood-2006-01-010215

[22] Chen VB, Arendall WB 3rd, Headd JJ, Keedy DA, Immormino RM, et al (2010) MolProbity: all-atom structure validation for macromolecular crystallography. Acta Crystallogr D Biol Crystallogr 66: 12–21. 20057044 10.1107/S0907444909042073 PMC2803126

[23] McGann M (2011) FRED pose prediction and virtual screening accuracy. J Chem Inf Model 51: 578–596. 21323318 10.1021/ci100436p

[24] Bostrom J, Greenwood JR, Gottfries J (2003) Assessing the performance of OMEGA with respect to retrieving bioactive conformations. J Mol Graph Model 21: 449–462. 12543140 10.1016/s1093-3263(02)00204-8

[25] Grynkiewicz G, Poenie M, Tsien RY (1985) A new generation of Ca2+ indicators with greatly improved fluorescence properties. The Journal of biological chemistry 260: 3440–3450. 3838314

[26] O'Connor MN, Smethurst PA, Farndale RW, Ouwehand WH (2006) Gain- and loss-of-function mutants confirm the importance of apical residues to the primary interaction of human glycoprotein VI with collagen. J Thromb Haemost 4: 869–873. 16405521 10.1111/j.1538-7836.2005.01764.x

[27] Smethurst PA, Joutsi-Korhonen L, O'Connor MN, Wilson E, Jennings NS, et al (2004) Identification of the primary collagen-binding surface on human glycoprotein VI by site-directed mutagenesis and by a blocking phage antibody. Blood 103: 903–911. 14504096 10.1182/blood-2003-01-0308

[28] Ono K, Ueda H, Yoshizawa Y, Akazawa D, Tanimura R, et al (2010) Structural basis for platelet antiaggregation by angiotensin II type 1 receptor antagonist losartan (DuP-753) via glycoprotein VI. J Med Chem 53: 2087–2093. 20158191 10.1021/jm901534d

[29] Grothusen C, Umbreen S, Konrad I, Stellos K, Schulz C, et al (2007) EXP3179 inhibits collagen-dependent platelet activation via glycoprotein receptor-VI independent of AT1-receptor antagonism: potential impact on atherothrombosis. Arterioscler Thromb Vasc Biol 27: 1184–1190. 17347483 10.1161/ATVBAHA.106.138693

[30] Murad JP, Espinosa EV, Ting HJ, Khasawneh FT (2011) Characterization of the In Vivo Antiplatelet Activity of the Antihypertensive Agent Losartan. J Cardiovasc Pharmacol Ther. 10.1177/1074248411425491 22123197

[31] Gibbins JM, Okuma M, Farndale R, Barnes M, Watson SP (1997) Glycoprotein VI is the collagen receptor in platelets which underlies tyrosine phosphorylation of the Fc receptor gamma-chain. Febs Letters 413: 255–259. 9280292 10.1016/s0014-5793(97)00926-5

[32] Poole A, Gibbins JM, Turner M, van Vugt MJ, van de Winkel JG, et al (1997) The Fc receptor gamma-chain and the tyrosine kinase Syk are essential for activation of mouse platelets by collagen. EMBO J 16: 2333–2341. 9171347 10.1093/emboj/16.9.2333 PMC1169834

[33] Murad JP, Espinosa EV, Ting HJ, Khasawneh FT (2012) Characterization of the in vivo antiplatelet activity of the antihypertensive agent losartan. J Cardiovasc Pharmacol Ther 17: 308–314. 22123197 10.1177/1074248411425491

[34] Guerra JI, Monton M, Rodriguez-Feo JA, Farre J, Jimenez AM, et al (2000) [Effect of losartan on human platelet activation by thromboxane A2]. Rev Esp Cardiol 53: 525–530. 10758030 10.1016/s0300-8932(00)75123-2

[35] Shoichet BK (2006) Screening in a spirit haunted world. Drug Discov Today 11: 607–615. 16793529 10.1016/j.drudis.2006.05.014 PMC1524586

[36] Julius S, Kjeldsen SE, Weber M, Brunner HR, Ekman S, et al (2004) Outcomes in hypertensive patients at high cardiovascular risk treated with regimens based on valsartan or amlodipine: the VALUE randomised trial. Lancet 363: 2022–2031. 15207952 10.1016/S0140-6736(04)16451-9

[37] Feher G, Feher A, Pusch G, Lupkovics G, Szapary L, et al (2009) The genetics of antiplatelet drug resistance. Clin Genet 75: 1–18. 19067731 10.1111/j.1399-0004.2008.01105.x

[38] Maree AO, Curtin RJ, Chubb A, Dolan C, Cox D, et al (2005) Cyclooxygenase-1 haplotype modulates platelet response to aspirin. J Thromb Haemost 3: 2340–2345. 16150050 10.1111/j.1538-7836.2005.01555.x

[39] Lepantalo A, Mikkelsson J, Resendiz JC, Viiri L, Backman JT, et al (2006) Polymorphisms of COX-1 and GPVI associate with the antiplatelet effect of aspirin in coronary artery disease patients. Thromb Haemost 95: 253–259. 16493486 10.1160/TH05-07-0516

[40] Varenhorst C, James S, Erlinge D, Brandt JT, Braun OO, et al (2009) Genetic variation of CYP2C19 affects both pharmacokinetic and pharmacodynamic responses to clopidogrel but not prasugrel in aspirin-treated patients with coronary artery disease. Eur Heart J 30: 1744–1752. 19429918 10.1093/eurheartj/ehp157 PMC2709885

[41] O'Donnell CJ, Larson MG, Feng D, Sutherland PA, Lindpaintner K, et al (2001) Genetic and environmental contributions to platelet aggregation: the Framingham heart study. Circulation 103: 3051–3056. 11425767 10.1161/01.cir.103.25.3051

[42] Massberg S, Konrad I, Bultmann A, Schulz C, Munch G, et al (2004) Soluble glycoprotein VI dimer inhibits platelet adhesion and aggregation to the injured vessel wall in vivo. Faseb J 18: 397–399. 14656994 10.1096/fj.03-0464fje

[43] Chen L, Gui C, Luo X, Yang Q, Gunther S, et al (2005) Cinanserin is an inhibitor of the 3C-like proteinase of severe acute respiratory syndrome coronavirus and strongly reduces virus replication in vitro. Journal of virology 79: 7095–7103. 15890949 10.1128/JVI.79.11.7095-7103.2005 PMC1112131

[44] Yang Q, Chen L, He X, Gao Z, Shen X, et al (2008) Design and synthesis of cinanserin analogs as severe acute respiratory syndrome coronavirus 3CL protease inhibitors. Chemical & pharmaceutical bulletin 56: 1400–1405. 18827378 10.1248/cpb.56.1400

[45] Bal Dit Sollier C, Crassard I, Simoneau G, Bergmann JF, Bousser MG, et al (2009) Effect of the thromboxane prostaglandin receptor antagonist terutroban on arterial thrombogenesis after repeated administration in patients treated for the prevention of ischemic stroke. Cerebrovascular diseases 28: 505–513. 19752552 10.1159/000236915

[46] Bousser MG, Amarenco P, Chamorro A, Fisher M, Ford I, et al (2011) Terutroban versus aspirin in patients with cerebral ischaemic events (PERFORM): a randomised, double-blind, parallel-group trial. Lancet 377: 2013–2022. 21616527 10.1016/S0140-6736(11)60600-4

[47] Belhassen L, Pelle G, Dubois-Rande JL, Adnot S (2003) Improved endothelial function by the thromboxane A2 receptor antagonist S 18886 in patients with coronary artery disease treated with aspirin. Journal of the American College of Cardiology 41: 1198–1204. 12679222 10.1016/s0735-1097(03)00048-2

[48] Lindholm LH, Ibsen H, Dahlof B, Devereux RB, Beevers G, et al (2002) Cardiovascular morbidity and mortality in patients with diabetes in the Losartan Intervention For Endpoint reduction in hypertension study (LIFE): a randomised trial against atenolol. Lancet 359: 1004–1010. 11937179 10.1016/S0140-6736(02)08090-X

[49] Kjeldsen SE, Dahlof B, Devereux RB, Julius S, Aurup P, et al (2002) Effects of losartan on cardiovascular morbidity and mortality in patients with isolated systolic hypertension and left ventricular hypertrophy: a Losartan Intervention for Endpoint Reduction (LIFE) substudy. Jama 288: 1491–1498. 12243636 10.1001/jama.288.12.1491

[50] Konstam MA, Neaton JD, Dickstein K, Drexler H, Komajda M, et al (2009) Effects of high-dose versus low-dose losartan on clinical outcomes in patients with heart failure (HEAAL study): a randomised, double-blind trial. Lancet 374: 1840–1848. 19922995 10.1016/S0140-6736(09)61913-9

[51] Kehoe PG, Passmore PA (2012) The renin-angiotensin system and antihypertensive drugs in Alzheimer's disease: current standing of the angiotensin hypothesis? J Alzheimers Dis 30 Suppl 2S251–268. 22330821 10.3233/JAD-2012-111376

